# Unenhanced computed tomography as a diagnostic tool in suspected pulmonary hypertension: a retrospective cross-sectional pilot study

**DOI:** 10.12688/wellcomeopenres.16853.2

**Published:** 2024-07-26

**Authors:** Ze Ming Goh, Christopher S. Johns, Tarik Julius, Samual Barnes, Krit Dwivedi, Charlie Elliot, Michael Sharkey, Dheyaa Alkanfar, Thanos Charalampololous, Catherine Hill, Smitha Rajaram, Robin Condliffe, David G. Kiely, Andrew J. Swift

**Affiliations:** 1 Department of Infection Immunity and Cardiovascular Disease, University of Sheffield Medical School, Sheffield, S10 2RX, UK; 2 Radiology Department, Sheffield Teaching Hospitals NHS Trust, Sheffield, S10 2JF, UK; 3 INSIGNEO, Institute of Insilico Medicine, Sheffield, S1 3JD, UK; 4 Sheffield Pulmonary Vascular Disease Unit, Sheffield Teaching Hospitals NHS Trust, Sheffield, S10 2JF, UK

**Keywords:** Computed tomography, Pulmonary hypertension, Diagnosis; Right Ventricle

## Abstract

**Background:**

Computed tomography pulmonary angiography (CTPA) has been proposed to be diagnostic for pulmonary hypertension (PH) in multiple studies. However, the utility of the unenhanced CT measurements diagnosing PH has not been fully assessed. This study aimed to assess the diagnostic utility and reproducibility of cardiac and great vessel parameters on unenhanced computed tomography (CT) in suspected pulmonary hypertension (PH).

**Methods:**

In total, 42 patients with suspected PH who underwent unenhanced CT thorax and right heart catheterization (RHC) were included in the study. Three observers (a consultant radiologist, a specialist registrar in radiology, and a medical student) measured the parameters by using unenhanced CT. Diagnostic accuracy of the parameters was assessed by area under the receiver operating characteristic curve (AUC). Inter-observer variability between the consultant radiologist (primary observer) and the two secondary observers was determined by intra-class correlation analysis (ICC).

**Results:**

Overall, 35 patients were diagnosed with PH by RHC while 7 patients were not. Main pulmonary arterial (MPA) diameter was the strongest (AUC 0.79 to 0.87) and the most reproducible great vessel parameter. ICC comparing the MPA diameter measurement of the consultant radiologist to the specialist registrar’s and the medical student’s were 0.96 and 0.92, respectively. Right atrial area was the cardiac measurement with highest accuracy and reproducibility (AUC 0.76 to 0.79; ICC 0.980, 0.950) followed by tricuspid annulus diameter (AUC 0.76 to 0.79; ICC 0.790, 0.800).

**Conclusions:**

MPA diameter and right atrial areas showed high reproducibility. Diagnostic accuracies of these were within the range of acceptable to excellent, and might have clinical value. Tricuspid annular diameter was less reliable and less diagnostic and was therefore not a recommended diagnostic measurement.

## Plain Language Summary

Pulmonary hypertension (PH) is a condition characterized by elevated pressure in the pulmonary artery and may lead to right heart failure. Several studies have demonstrated the diagnostic value of non-invasive techniques computed tomography (CT) with contrast in identifying PH. Therefore, we aim to investigate the diagnostic accuracy of non-contrast CT, which is commonly performed in patients with suspected lung diseases who are at risk of PH.

## Introduction

Pulmonary hypertension (PH) is defined by elevated resting mean pulmonary artery pressure (mPAP) at right heart catheterization (RHC) ^1– 3^. Poor prognosis is associated with the presence of pulmonary hypertension leading to the signs of right heart failure ^4^.

Pulmonary hypertension is categorised into five major groups in the European Society of Cardiology (ESC) and the European Respiratory Society (ERS) Guidelines ^1^. Group 1 (pulmonary arterial hypertension) is characterised by small vessel pulmonary arterial remodeling ^5, 6^, while group 2 is typically due to the passive backflow of blood into the lungs in the presence of left heart disease. Group 3 pulmonary hypertension is associated with lung disease or hypoxia, group 4 pulmonary hypertension is chronic thromboembolic pulmonary hypertension, and group 5 is caused by unclear multifactorial mechanisms including chronic haemolytic anaemia, sarcoidosis, thyroid disease and Gaucher disease ^5^. Group 1 pulmonary hypertension is a rare but life-limiting condition ^7^. Group 2 and 3 are the most prevalent forms of pulmonary hypertension and are associated with various cardiorespiratory diseases ^8^.

Transthoracic Doppler echocardiography (TTE) is a non-invasive test that is used to predict the right ventricular systolic pressure and diagnose pulmonary hypertension ^9– 12^. In a meta-analysis, the pooled sensitivity was 88% while the specificity was 56% ^13^. However, TTE is limited to only assess the right ventricle due to its shape and orientation. Views of the tricuspid regurgitant jet and cardiac chambers may be insufficient in patients with obesity or severe lung diseases ^14, 15^. Furthermore, accurate estimation of pulmonary arterial pressure could be challenging on TTE. For example, systolic pulmonary arterial pressure (sPAP) may be underestimated in patients with pronounced enlargement of regurgitating tricuspid orifice, or patients with a small amount of tricuspid regurgitation, particularly in those with chronic obstructive pulmonary disease (COPD) or other severe lung diseases. An overestimation of the right atrial pressure by echocardiographer could also lead to inaccurate estimation of sPAP. Other causes of inaccuracy include female gender, cardiac arrhythmia, systemic hypertension and diuretic.

Right heart catheterization (RHC) is known to be the gold standard diagnostic test for patients with suspected pulmonary hypertension ^1^. The morbidity and mortality of the procedure are 1% and 0.05%, respectively when being performed at a pulmonary hypertension centre ^16^. Although a relative low risk of mortality and morbidity are associated with the procedure, the invasive nature of RHC may lead to complications, such as pneumothoraces, arrhythmias and hypotensive episodes ^16^.

Computed tomography (CT) is a widespread imaging investigation in patients with unexplained breathlessness. It has been proposed that a measurement of the main pulmonary artery (MPA) diameter of 29 mm or larger on CT pulmonary angiography (CTPA) is diagnostic for pulmonary hypertension, with a sensitivity of 87% and a specificity of 89% ^17^. A ratio of MPA diameter to the adjacent ascending aorta that is more than 1 is suggested to be associated with pulmonary hypertension ^18^. Furthermore, the addition of measurement of the right ventricle on CT pulmonary angiography improves diagnostic accuracy ^19, 20^.

The majority of the past studies focus on the use of CTPA in diagnosing pulmonary hypertension, and MPA diameter has been proposed to be a reliable diagnostic criterion in multiple studies ^21^. Unenhanced CT thorax studies are commonly performed in patients with suspected lung disease. However, the utility of the unenhanced CT measurements of cardiac structures in diagnosing PH is not typically assessed due to the lack of contrast to outline the cardiac chambers. We hypothesise that measurements made on unenhanced CT studies using the visible landmarks are reproducible and have significant diagnostic value.

This study aims to determine (a) the diagnostic value and (b) reproducibility of the measurements of cardiac and great vessel structures on unenhanced CT thorax studies.

## Methods

The patients were selected through database search of the ASPIRE (Assessing the Spectrum of Pulmonary Hypertenison Identified at a Referral Centre) Registry. Patients with suspected pulmonary hypertension who were referred to the Royal Hallamshire Hospital, Sheffield and underwent baseline unenhanced (CT) thorax or high-resolution CT (HRCT) within 90 days of right heart catheterization between 23 May 2012 to 15 January 2016 were included in the study. The term used for searching was ‘CT thorax’ and the images were checked visually to ensure no contrast occured. Unenhanced CT images were acquired in routine clinical practice.

### Ethics statement

Ethical approval by the North Sheffield Ethics Committee and review board was obtained (reference c06/Q2308/8) for the study. Written patient consent was not required due to the retrospective nature of the study. Consent for participation and publication was waived by the Sheffield Teaching Hospital NHS Foundation Trust (STH19500). 

### CT image acquisition

Unenhanced CT scans carried out in routine clinical practice were identified, from review of the ASPIRE registry database. Inclusion criteria used were diagnostic imaging and thoracic CT with full lung coverage. Records of patients who fulfilled the inclusion criteria were reviewed individually. Both volumetric acquisition and high-resolution CT were included in the analysis. Unenhanced CT studies were conducted in Royal Hallamshire Hospital or at the patient’s local hospital prior to referral. All studies in Royal Hallamshire Hospital were performed on a 64-slice MDCT scanner (light-speed General Electric Medical Systems, Milwaukee, WI). Slice thickness was less than or equal to 5 mm for inclusion. Criteria for exclusion included patients who were not diagnosed with pulmonary hypertension after RHC and had not been assessed with unenhanced CT. 

The CT imaging acquisition parameters were as follows: 100mA with automated dose reduction, 120kV, pitch 1, rotation time 0.5s and 1 mm collimation. A 400 mm×400 mm field of view was used with an acquisition matrix of 512×512. HRCT were reconstructed using the contrast-enhanced acquisitions with 1.25 mm collimation from the apex of the lung to the diaphragm. The reconstruction kernel used was lung standard volume with FC53.

### Image analysis

**Figure 1.**
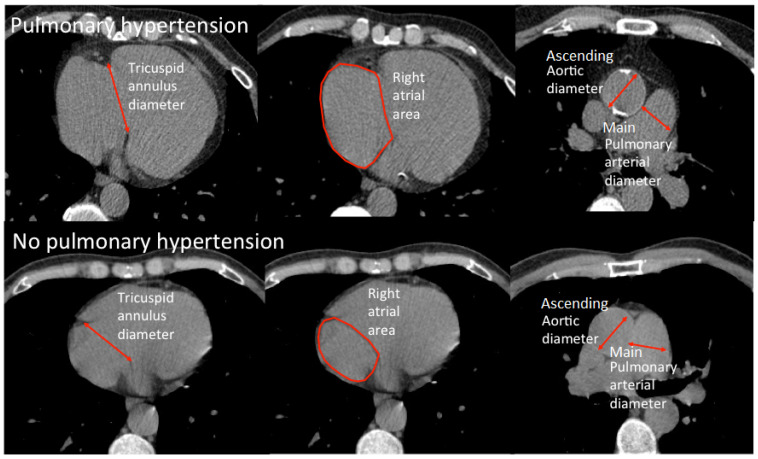
Image of measurements of tricuspid annulus diameter, right atrial area, ascending aortic diameter and main pulmonary arterial diameter in patients with and without pulmonary hypertension.

Three observers (a consultant radiologist (AJS), a specialist registrar in radiology (TJ) and a medical student (ZMG)) recorded measurements of the great vessels and cardiac structures. The observers were blinded to all the other clinical and imaging data. All observers were also blinded to each other’s results and the results of the right heart catheterisation. The measurements were carried out on axial images. Unenhanced CT measurements of the vessels included MPA diameter, the diameter of the ascending aorta, right and left pulmonary arterial diameter and diameter of the superior vena cava. MPA diameter was measured as the maximal perpendicular diameter of the main pulmonary artery before the bifurcation. Maximal diameter of ascending aorta was measured. Right and left pulmonary arterial diameters were measured at the widest portion distal to the bifurcation. Maximal diameter of superior vena cave was measured. On the other hand, the cardiac measurements included tricuspid annular diameter, mitral annular diameter, left and right atrial area. Tricuspid and mitral annular diameters were measured as the maximal diameter of each annulus. Left and right atrial areas were measured as the maximal area of the left and right atria, respectively. All the images were measured manually on axial sections in mediastinal window settings. Figure 1 illustrates how the measurements of tricuspid annulus diameter, right atrial area, ascending aortic diameter and MPA diameter were made on unenhanced CT images. New variables were derived from the measured variables. The ratio of the MPA diameter to the diameter of ascending aorta (MPA/AAo) and the body surface area (MPA/BSA) were calculated. Other derived variables included the ratio of right and left pulmonary arterial diameter to the diameter of the ascending aorta as well as the ratio of the tricuspid annular diameter to the mitral annular diameter.

### Statistics

All statistic analyses were carried out using the IBM SPSS Statistic 26. PSPP is an open access alternative to SPSS that could be used to perform the same analysis. Pearson’s correlation test was used to identify measured and derived variables that had significant correlation with mPAP. T-tests were performed for all measured and derived variables. Group comparisons between patients with pulmonary hypertension and without pulmonary hypertension were made using independent T-test. The variables were considered significant for t-test when p < 0.05.

All of the measured and derived variables were also tested with receiver operating characteristic (ROC) curve test to identify variables that might be useful in diagnosing pulmonary hypertension. The thresholds used to evaluate the diagnostic accuracy of the variables were as follows: AUC of 0.5 suggested no discrimination, 0.7 to 0.8 was considered acceptable, 0.8 to 0.9 was deemed to be excellent and more than 0.9 was considered outstanding ^22^. 

Image analysis was carried out independently by 3 observers (a consultant radiologist, a specialist radiologist and a medical student). Intra-class correlation coefficient (ICC) test was then used to assess the reproducibility by comparing the result of the consultant radiologist with the results of the specialist registrar and medical student.

## Results

### Patients

A demographic table of the population of the study was produced ( Table 1). A Total of 42 incident patients with suspected pulmonary hypertension that underwent unenhanced (CT) thorax and right heart catheterization (RHC) were identified ^23^. There was no missing data of unenhanced CT measurements of the patients. Of those, 35 patients were diagnosed with pulmonary hypertension through RHC while 7 patients were not. The mean age of the pulmonary hypertensive and non-pulmonary hypertensive groups were 67 (SD 11) and 63 (SD 12), respectively. There was no significant difference in age between the two groups (p=0.391). 86% of the sample was sourced from hospitals in Sheffield.

**Table 1.** Demographic table which contains mean, standard deviation and p-values of different variables. The measurements are based on results of the consultant radiologist.

	With Pulmonary hypertension (n=35)		Without Pulmonary Hypertension (n=7)		
Variables	Mean	Standard Deviation	Mean	Standard Deviation	p-value
Range					
Age (years)	67.4	10.9	63.4	11.8	0.391
Sex	Male=18 Female=17		Male=5 Female=2		
WHO Functional Class	3.2	0.4	2.7	0.5	0.007
Right Heart Catheterization					
Pulmonary Wedge Pressure	15	5	9	3	0.007
Mean Right Atrial Pressure	12	6	5	2	0.007
Mean Pulmonary Arterial Pressure	46	13	19	3	0.000
Cardiac Output	4.9	1.2	6.2	1.7	0.022
Pulmonary Vascular Resistance	579	378	140	69	0.000
SVo2	64	8	70	7	0.043
CT (Unenhanced)					
Main Pulmonary Arterial Diameter (mm)	34	5	29	8	0.049
Tricuspid Annulus Diameter (mm)	50	7	44	5	0.025
Mitral Annulus Diameter (mm)	37	6	36	4	0.528
Tricuspid Annulus Diameter: Mitral Annulus Diameter	1.4	0.3	1.2	0.2	0.214
Right Atrial Area (mm ^2^)	3043	1124	1900	579	0.001
Left Atrial Area (mm ^2^)	2619	1127	1718	334	0.000
Diameter of the ascending aorta (mm)	35	5	33	5	0.356
Right Pulmonary Arterial Diameter (mm)	27	4	23	5	0.047
Left Pulmonary Arterial Diameter (mm)	26	3	23	5	0.116
MPA/AAo ^2^	1.0	0.2	0.9	0.1	0.175
RPA/AAo ^3^	0.8	0.2	0.7	0.2	0.497
LPA/AAo ^4^	0.8	0.1	0.7	0.2	0.668
MPA/BSA ^5^	18.6	3.3	16.2	3.6	0.096
Superior Vena Cava Area (mm ^2^)	337	138	286	45	0.091

^1^Mixed venous oxygen saturation ^2^Ratio of the main pulmonary arterial (MPA) diameter to the diameter of ascending aorta (AAo) ^3^Ratio of the right pulmonary arterial (RPA) diameter to the diameter of ascending aorta (AAo) ^4^Ratio of the left pulmonary arterial (LPA) diameter to the diameter of ascending aorta (AAo) ^5^Ratio of the main pulmonary arterial (MPA) diameter to the body surface area (BSA)

### Correlations

Pearson correlations ( Table 2) were calculated against mPAP for all related variables of CT measurements. Based on the results of the consultant radiologist, right atrial area (r=0.48, p=0.001), tricuspid annulus diameter (r=0.36, p=0.02), and the MPA/AAo (r=0.40, p=0.008), showed the strongest association with mPAP. MPA diameter had a weaker correlation with mPAP (r=0.21, p=0.186) than MPA/AAo. Only right atrial area and MPA/AAo were found to have moderate correlation against mPAP (0.4-0.6). Most correlations were modest (0.2-0.4).

**Table 2.** Pearson Correlations of all variables to mean pulmonary artery pressure based on data of the consultant radiologist, specialist registrar and medical student, including r values and p values.

	Consultant Radiologist		Specialist Registrar		Medical Student	
Variables	r Value	p Value	r Value	p Value	r Value	p Value
Main Pulmonary Arterial Diameter (mm)	0.208	0.186	0.242	0.122	0.292	0.061
Tricuspid Annulus Diameter (mm)	0.358	0.02	0.363	0.018	0.170	0.287
Mitral Annulus Diameter (mm)	0.061	0.699	0.35	0.023	0.052	0.744
Tricuspid Annulus Diameter: Mitral Annulus Diameter	0.236	0.132	0.072	0.650	-0.367	0.017
Right Atrial Area (mm ^2^)	0.475	0.001	0.353	0.022	0.391	0.010
Left Atrial Area (mm ^2^)	0.192	0.222	0.356	0.021	0.170	0.282
Diameter of the ascending aorta (mm)	-0.19	0.228	-0.135	0.394	-0.18	0.253
Right Pulmonary Arterial Diameter (mm)	0.231	0.142	0.276	0.076	0.149	0.346
Left Pulmonary Arterial Diameter (mm)	0.162	0.305	0.256	0.102	0.051	0.749
MPA/AAo ^1^	0.403	0.008	0.444	0.003	0.444	0.003
RPA/AAo ^2^	0.306	0.490	0.441	0.003	0.332	0.032
LPA/AAo ^3^	0.295	0.058	0.347	0.024	0.249	0.112
MPA/BSA ^4^	0.179	0.258	0.231	0.141	0.220	0.162
Superior Vena Cava Area(mm ^2^)	0.126	0.425	0.319	0.04	0.223	0.155

^1^Ratio of the main pulmonary arterial (MPA) diameter to the diameter of ascending aorta (AAo) ^2^Ratio of the right pulmonary arterial (RPA) diameter to the diameter of ascending aorta (AAo) ^3^Ratio of the left pulmonary arterial (LPA) diameter to the diameter of ascending aorta (AAo) ^4^Ratio of the main pulmonary arterial (MPA) diameter to the body surface area (BSA)

### Diagnostic accuracy

**Figure 2.**
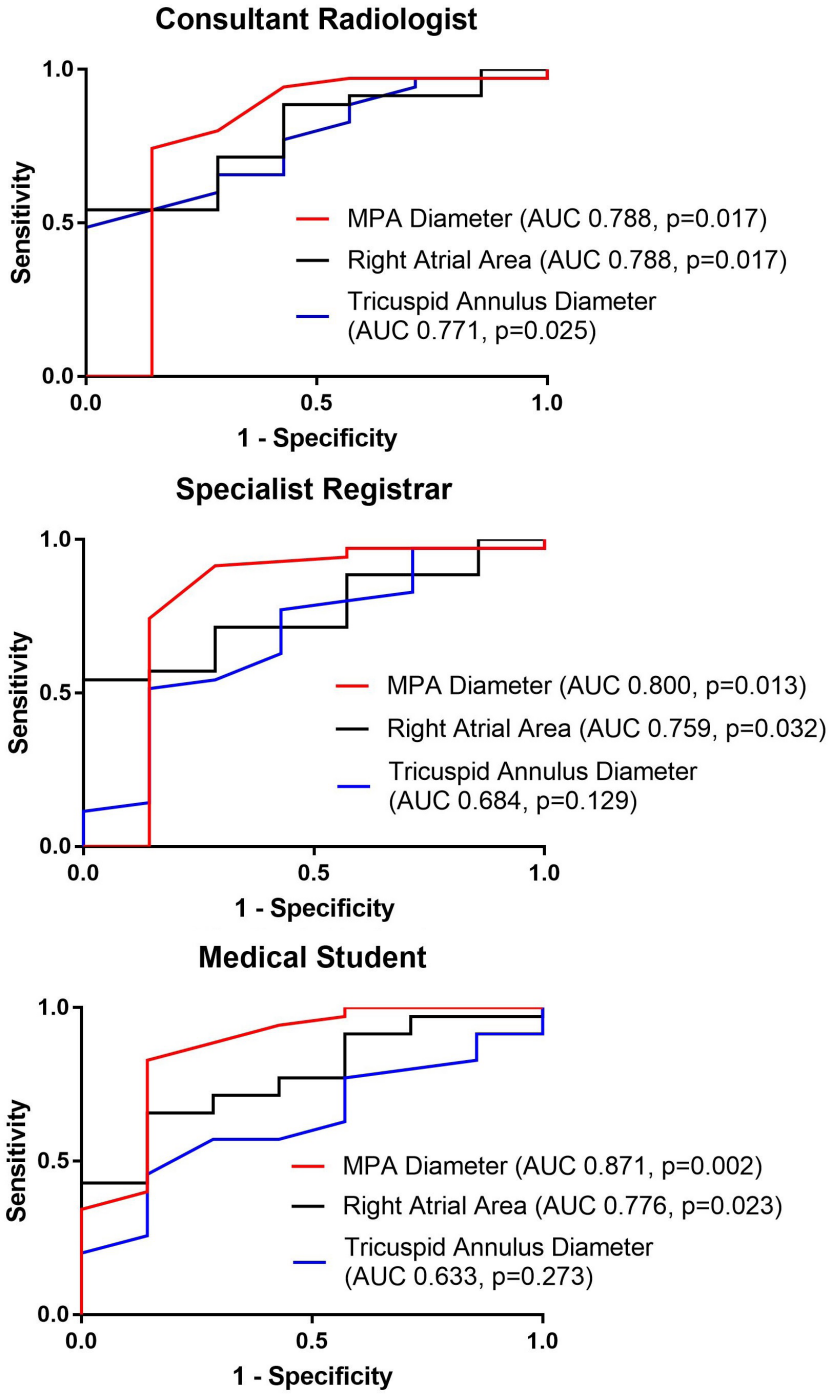
Receiver operating characteristic (ROC) curves of main pulmonary arterial (MPA) diameter, right atrial area and tricuspid annulus diameter for predicting presence of pulmonary hypertension; and respective area under the receiver operating characteristic curve (AUC), and p-values.

The results of the T-tests were significant (p<0.05) for MPA diameter, right atrial area and tricuspid annulus diameter. ROC curve of MPA diameter, right atrial area and tricuspid annulus diameter were plotted ( Figure 2). AUC of all measurements were recorded ( Table 3). MPA diameter and right atrial area consistently showed to have AUC of more than 0.7 in all 3 observers’ results. AUC values of MPA diameter were 0.79 (consultant radiologist), 0.80 (specialist registrar) and 0.87 (medical student). The result demonstrated that the diagnostic accuracy of MPA diameter ranged from acceptable or nearly excellent (the lowest AUC value was very close to the threshold to be considered as excellent) to excellent. AUC values of right atrial area were 0.79 (consultant radiologist), 0.76 (specialist registrar) and 0.78 (medical student). It was shown that right atrial area had a diagnostic accuracy that was considered to be acceptable. Weaker accuracy was found for MPA/BSA (AUC 0.65 to 0.77) and MPA/AAo (AUC 0.65 to 0.69). Right atrial area and tricuspid annulus diameter were the strongest cardiac measurements, AUC 0.76 to 0.79, and AUC 0.63 to 0.77, respectively.

**Table 3.** Area under the receiver operating characteristic curve (AUC) of all measurements for predicting presence of pulmonary hypertension.

	Consultant Radiologist				Specialist Registrar				Medical Student			
Variables	AUC	p-value	95% Confidence Interval		AUC	p-value	95% Confidence Interval		AUC	p-value	95% Confidence Interval	
			Lower Bound	Upper Bound			Lower Bound	Upper Bound			Lower Bound	Upper Bound
Tricuspid Annulus Diameter	0.771	0.025	0.608	0.935	0.684	0.129	0.459	0.908	0.633	0.273	0.436	0.829
Mitral Annulus Diameter	0.565	0.589	0.356	0.775	0.739	0.048	0.553	0.925	0.580	0.510	0.325	0.835
Right Atrial Area	0.788	0.017	0.628	0.948	0.759	0.032	0.601	0.918	0.776	0.023	0.610	0.941
Left Atrial Area	0.784	0.019	0.615	0.953	0.694	0.109	0.513	0.875	0.735	0.052	0.562	0.907
Main ulmonary Arterial (MPA) Diameter	0.788	0.017	0.537	1.000	0.800	0.013	0.549	1.000	0.871	0.002	0.707	1.000
Right Pulmonary Arterial Diameter (RPA)	0.761	0.031	0.512	1.000	0.869	0.002	0.757	0.982	0.800	0.013	0.549	1.000
Left Pulmonary Arterial Diameter (LPA)	0.720	0.068	0.473	0.968	0.743	0.045	0.496	0.990	0.688	0.121	0.400	0.975
Ascending Aortic Diameter (AAo)	0.624	0.303	0.384	0.865	0.702	0.095	0.491	0.913	0.635	0.265	0.419	0.850
Superior Vena Cava Area	0.571	0.555	0.404	0.739	0.698	0.102	0.545	0.851	0.690	0.117	0.538	0.841
Tricuspid Annulus Diameter: Mitral Annulus Diameter	0.627	0.295	0.404	0.849	0.445	0.649	0.227	0.662	0.531	0.800	0.321	0.740
MPA/AAo ^1^	0.694	0.109	0.487	0.901	0.645	0.231	0.412	0.878	0.690	0.117	0.480	0.899
RPA/AAo ^2^	0.618	0.328	0.337	0.899	0.676	0.147	0.440	0.911	0.655	0.200	0.423	0.888
LPA/AAo ^3^	0.653	0.206	0.380	0.926	0.620	0.319	0.355	0.886	0.561	0.613	0.309	0.814
MPA/BSA ^4^	0.653	0.206	0.418	0.888	0.676	0.147	0.450	0.901	0.765	0.028	0.599	0.932

^1^Ratio of the main pulmonary arterial (MPA) diameter to the diameter of ascending aorta (AAo) ^2^Ratio of the right pulmonary arterial (RPA) diameter to the diameter of ascending aorta (AAo) ^3^Ratio of the left pulmonary arterial (LPA) diameter to the diameter of ascending aorta (AAo) ^4^Ratio of the main pulmonary arterial (MPA) diameter to the body surface area (BSA)

The present study displayed that MPA diameter had a sensitivity of 80% (28/35) and specificity of 71% (5/7) when adopting a threshold of >29 mm to diagnose pulmonary hypertension and had a sensitivity of 94% (33/35) and specificity of 57% (4/7) when using >27 mm as the threshold. diagnosing pulmonary hypertension with the threshold of MPA/AAo > 1 had a sensitivity of 34% (12/35) and a specificity of 86% (6/7). The sensitivity and specificity of right atrial area were 57% (20/35) and 71% (5/7), respectively when using a threshold of >2500 mm ^2^. Using the previously defined >40 mm threshold in the echocardiography literature ^24^, the tricuspid annulus diameter had sensitivity and specificity of 94% (33/35) and 29% (2/7) respectively.

### Reproducibility

Table 4 demonstrates the result of the ICC test when comparing measurements of the consultant radiologist with the measurements of the specialist registrar and medical student. MPA diameter was the most reproducible great vessel measurement; ICC comparing the consultant radiologist with the specialist registrar and the medical student were 0.960 and 0.916, respectively. Right atrial area was the cardiac metric with the highest reproducibility, 0.980 and 0.950, respectively. Tricuspid annulus diameter showed good reproducibility of 0.790 and 0.800, respectively.

**Table 4.** Reproducibility tests of the variables including 95% confidence interval and p values. Data correlated between the consultant radiologist against the specialist registrar and medical students.

Variable	Results of the Consultant Radiologist					
	Against Results of the Specialist Registrar			Against Results of the Medical Students		
	Average measures	95% CI Lower	95% CI Upper	Average measures	95% CI Upper	95% CI Lower
Tricuspid Annulus Diameter	0.794	0.617	0.889	0.799	0.604	0.887
Mitral Annulus Diameter	0.720	0.480	0.850	0.699	0.441	0.838
Right Atrial Area	0.975	0.953	0.986	0.945	0.897	0.97
Left Atrial Area	0.980	0.897	0.970	0.888	0.792	0.94
Pulmonary Arterial diameter (PA)	0.964	0.933	0.981	0.916	0.843	0.955
Right Pulmonary Arterial Diameter (RPA)	0.895	0.805	0.944	0.834	0.691	0.911
Left Pulmonary Arterial Diameter (LPA)	0.936	0.881	0.966	0.745	0.525	0.863
Ascending Aorta Diameter (AAo)	0.922	0.854	0.958	0.824	0.672	0.905
Superior Vena Cava Area	0.895	0.804	0.944	0.817	0.659	0.901
Tricuspid Annulus Diameter/Mitral Annulus Diameter	0.720	0.480	0.850	0.727	0.493	0.853
MPA/AAo ^1^	0.830	0.683	0.908	0.655	0.358	0.815
RPA/AAo ^2^	0.822	0.669	0.904	0.649	0.347	0.811
LPA/AAo ^3^	0.777	0.585	0.880	0.302	-0.299	0.625
MPA/BSA ^4^	0.966	0.937	0.982	0.944	0.896	0.97

^1^Ratio of the main pulmonary arterial (MPA) diameter to the diameter of ascending aorta (AAo) ^2^Ratio of the right pulmonary arterial (RPA) diameter to the diameter of ascending aorta (AAo) ^3^Ratio of the left pulmonary arterial (LPA) diameter to the diameter of ascending aorta (AAo) ^4^Ratio of the main pulmonary arterial (MPA) diameter to the body surface area (BSA)

## Discussion

This pilot study has demonstrated that simple unenhanced CT measurements such as MPA diameter and right atrial area have diagnostic value in cases of suspected pulmonary hypertension. This data may be of value for powering a definitive trial to assess the value of unenhanced CT for the diagnosis of pulmonary hypertension. This data requires confirmation in larger definitive trials in the setting of a tertiary referral centre but also in screening populations, for example, patients undergoing unenhanced CT thorax or HRCT for unexplained breathlessness or assessment of lung disease. 

There are several clinical advantages of using unenhanced CT as a diagnostic tool when pulmonary hypertension is suspected. Firstly, unenhanced CT is commonly used in patients with suspected parenchymal lung disease who are susceptible to develop pulmonary hypertension. Hence, these patients can be assessed for pulmonary hypertension at the same time, and the need for further investigation could be determined. Secondly, patients who are at risk could be identified through unenhanced CT and be referred on for echocardiography. These include patients who receive unenhanced CT scans through other referral criteria such as assessment of emphysema, lung fibrosis or bronchiectasis. Thus, a more prompt diagnosis of pulmonary hypertension could be made. The application of deep learning techniques on common imaging modalities including CT has been widely studied ^25^. Incorporation of the techniques with diagnostic markers of pulmonary hypertension on CT images could potentially assist physicians in making early diagnosis with greater accuracy.

According to a research carried out by The Framingham Heart Study, the 90 ^th^ percentile sex-specific cutoff values of normal MPA diameter were 29 mm in men and 27 mm in women ^26^. The study also showed that the participants who were in sex-specific 90 ^th^ percentile group (men = 28.0 – 29.2 mm women = 26.6 – 27.4 mm) were associated with an increased risk of self-reported dyspnoea ^26^. A study conducted by Tan *et al*. suggested that MPA diameter of ≥29 mm had a sensitivity of 87% and specificity of 89% ^17^. The present study displayed that MPA diameter had a sensitivity of 80% (28/35) and specificity of 71% (5/7) when adopting a threshold of >29 mm to diagnose pulmonary hypertension and had a sensitivity of 94% (33/35) and specificity of 57% (4/7) when using >27 mm as the threshold. 

The ratio of MPA diameter to the diameter of ascending aorta (MPA/AAo) >1 has been found to be 70% sensitive and 92% specific for mPAP >20 mmHg ^18^. Besides, Sanal *et al*. demonstrated that MPA/AAo ≥ 1 have moderate diagnostic accuracy of 59% sensitivity and 82% specificity ^27^. In the present study, the sensitivity and specificity of MPA/AAo >1 were 34% (12/35) and 86% (6/7), respectively. Therefore, the results of the present study reflected that MPA diameter was a better indicator to be used in assessing pulmonary hypertension compared to MPA/AAo. 

Multiple right ventricular and pulmonary arterial measurements have been identified to be diagnostic on cardiac magnetic resonance imaging (MRI) ^6, 7, 28– 30^. Right cardiac structures such as right atrial area and tricuspid annulus diameter were not assessed previously on unenhanced CT for the diagnosis of pulmonary hypertension. Besides, radiologists usually do not assess the parameter of cardiac structures on unenhanced CT scans due to the lack of visible landmarks. Based on the results of the present study, right atrial area (AUC 0.76 to 0.79) was potentially a useful diagnostic parameter and had better diagnostic accuracy than tricuspid annulus diameter (AUC 0.63 to 0.77). Tricuspid annulus diameter was less diagnostic and had less clinical value in diagnosing pulmonary hypertension. Furthermore, left atrial area demonstrated comparable diagnostic accuracy (AUC 0.74 to 0.78) to right atrial area. This could possibly be explained by the inclusion of patients with pulmonary hypertension due to left heart disease in the study, as they are likely to have left atrial dilatation. More studies were required to investigate the clinical utilities of these right cardiac structures in suspected pulmonary hypertension, including their screening and diagnostic accuracies.

## Reproducibility

In the present study, measurements of structures that were useful in the diagnosis of suspected pulmonary hypertension showed to have high reproducibility, especially MPA diameter and right atrial area. Currently, there are limited studies that have been carried out to assess the reproducibility of these structures.

Measurements of these structures proved to be useful even in the absence of intravenous contrast. The results were reproducible despite the lack of anatomical landmarks on the unenhanced CT scans and differences in the amount of experience between the observers. Therefore, it was demonstrated that a vast amount of experience in analysing radiological images was not necessarily required to assess for pulmonary hypertension by using unenhanced CT.

## Limitations

The retrospective study design and the small number of patients included were limitations of the study. Furthermore, the number of patients in the non-pulmonary hypertensive group was relatively few. However, this pilot study is representative of a tertiary referral population with suspected pulmonary hypertension. Further work is required to evaluate the diagnostic value in routine reporting of unenhanced CT thorax or HRCT scans whether the proportion of patients without pulmonary hypertension is far higher. The lack of comparison with CT pulmonary angiography was also a limitation. The lack of cardiac gating was another limitation, leading to variability in pulmonary arterial and cardiac measurements. However, given that cardiac gating is not typically employed for unenhanced CT thorax or HRCT scans, the results of the present study are clinically relevant. During the 6 ^th^ World Symposium on Pulmonary Hypertension in 2018, it was recommended to diagnose pulmonary hypertension using a threshold of mPAP >20 mmHg and pulmonary vascular resistance >3 Wood units ^31^. However, the present study in keeping with current ESC/ERS guidelines has adopted the previous diagnostic threshold of mPAP ≥ 25 mmHg and pulmonary vascular resistance >3 Wood units ^1^. Further work to assess the accuracy of unenhanced CT in larger populations to identify optimal cut offs for CT parameters according to the World symposium threshold is required ^31^. The diagnostic thresholds identified for right heart structures (tricuspid annulus threshold and the right atrial threshold based on echocardiography and magnetic resonance imaging literature) would require validation in further cohorts of patients with suspected pulmonary hypertension.

## Conclusions

MPA diameter and right atrial areas showed high reproducibility when being measured on the unenhanced CT scans. Diagnostic accuracy of these in patients with suspected pulmonary hypertension were within the range of acceptable to excellent, and might have clinical value. Tricuspid annular diameter was less reliable and less diagnostic and was therefore not a recommended diagnostic measurement. Further studies in larger tertiary referral populations and screening populations that aimed to evaluate the diagnostic value of MPA diameter and right atrial area in patients with suspected pulmonary hypertension were advised.

## Data Availability

Figshare: Data. https://doi.org/10.6084/m9.figshare.16527519.v3 ^23^. This project contains the following underlying data: -  Data.xlsx (imaging measurements of cardiac structures and great vessels in patients with and without pulmonary hypertension). Data are available under the terms of the Creative Commons Attribution 4.0 International license (CC-BY 4.0).
