# Supplementary material for: Unenhanced computed tomography as a diagnostic tool in suspected pulmonary hypertension: a retrospective cross-sectional pilot study
Source: Wellcome Open Res. 2024 Jul 26;6:249. Originally published 2021 Sep 29. [Version 2] doi: 10.12688/wellcomeopenres.16853.2 (PMC11303945; doi:10.12688/wellcomeopenres.16853.2)
Supplement: Peer review history

# Reviewer report: Reviewer response for version 2

**DOI:** 10.21956/wellcomeopenres.25168.r91055

**Published:** 2024-08-06

**Authors:** Joseph Jacob

**Affiliations:** 1 Centre for Medical Image Computing, University College London, London, UK

Happy with all the changes made.

Is the work clearly and accurately presented and does it cite the current literature?

Partly

If applicable, is the statistical analysis and its interpretation appropriate?

Partly

Are all the source data underlying the results available to ensure full reproducibility?

No source data required

Is the study design appropriate and is the work technically sound?

Yes

Are the conclusions drawn adequately supported by the results?

Yes

Are sufficient details of methods and analysis provided to allow replication by others?

Yes

Reviewer Expertise:

CT and CXR imaging of the lungs

I confirm that I have read this submission and believe that I have an appropriate level of expertise to confirm that it is of an acceptable scientific standard.

# Reviewer report: Reviewer response for version 2

**DOI:** 10.21956/wellcomeopenres.25168.r91054

**Published:** 2024-08-06

**Authors:** Andrea Sonaglioni

**Affiliations:** 1 Istituto di Ricovero e Cura a Carattere Scientifico (IRCCS) MultiMedica, Milan, Italy

The Authors satisfied my suggestions. The manuscript is considerably improved. I do not have other suggestions.

Is the work clearly and accurately presented and does it cite the current literature?

Yes

If applicable, is the statistical analysis and its interpretation appropriate?

Yes

Are all the source data underlying the results available to ensure full reproducibility?

Yes

Is the study design appropriate and is the work technically sound?

Yes

Are the conclusions drawn adequately supported by the results?

Yes

Are sufficient details of methods and analysis provided to allow replication by others?

Yes

Reviewer Expertise:

Echocardiography, speckle tracking echocardiography, heart failure, valvular heart disease, mitral valve prolapse, pectus excavatum, pulmonary hypertension

I confirm that I have read this submission and believe that I have an appropriate level of expertise to confirm that it is of an acceptable scientific standard.

# Reviewer report: Reviewer response for version 1

**DOI:** 10.21956/wellcomeopenres.18590.r88546

**Published:** 2024-07-15

**Authors:** Andrea Sonaglioni

**Affiliations:** 1 Istituto di Ricovero e Cura a Carattere Scientifico (IRCCS) MultiMedica, Milan, Italy

The Authors demonstrated that MPA diameter and right atrial area measured on unenhanced CT scan have an acceptable to excellent diagnostic accuracy for predicting pulmonary hypertension detected on RHC.

 The Authors found that a MPA diameter ≥29 mm had the best sensitivity and specificity for noninvasively identifying patients with suspected pulmonary hypertension.

 The text is well written, each section is clearly presented, the statistics is adequate, the tables and figures are interesting and the discussion is complete.

 The strength of the manuscript is the proposal of a noninvasive technique, such as unenhanced CT scan, to predict pulmonary hypertension.

**I have some suggestions for the Authors:**

- In the Introduction section, there are some refusals: line 8, please delete the double “the” “the”.
- In the test body please correct the refusals: 29mm and replace with 29 mm.
- In the Methods section, please correct the refusal “imaiges” and replace with “images”.
- In the Introduction section, the Authors could expand the limitations of transthoracic echocardiography in the systolic pulmonary artery pressure (sPAP) estimation.

Notably, sPAP may be underestimated: in the presence of pronounced enlargement of the effective area of the regurgitating tricuspid oriﬁce, which causes the reduction of tricuspid regurgitation velocity (TRV) and ‘truncation’ of the CW-Doppler spectrum of tricuspid regurgitation; in case of a small amount of tricuspid regurgitation leading to an incomplete Doppler spectrum, with consequent underestimation of SPAP, especially in patients with COPD and/or advanced lung disease.

 On the other hand, sPAP may not be accurate due to the frequent overestimation of right atrial pressure by echocardiographers.

 Other causes of TTE imprecision in estimating sPAP may be the following: female gender, arrhythmic cardiac electrical activity, systemic arterial hypertension, and diuretic treatment.

 With this regard, the Authors could cite and discuss the following references: Finkelhor *et al.* (2015 ^1^), Kyranis *et al.* (2018 ^2^) and Sonaglioni *et al.* (2021 ^3^).

 In the Limitations section, the Authors could also mention that CT scan is limited by the ionizing exposition.

Is the work clearly and accurately presented and does it cite the current literature?

Yes

If applicable, is the statistical analysis and its interpretation appropriate?

Yes

Are all the source data underlying the results available to ensure full reproducibility?

Yes

Is the study design appropriate and is the work technically sound?

Yes

Are the conclusions drawn adequately supported by the results?

Yes

Are sufficient details of methods and analysis provided to allow replication by others?

Yes

Reviewer Expertise:

Echocardiography, speckle tracking echocardiography, heart failure, valvular heart disease, mitral valve prolapse, pectus excavatum

I confirm that I have read this submission and believe that I have an appropriate level of expertise to confirm that it is of an acceptable scientific standard, however I have significant reservations, as outlined above.

## References

[1] : Limitations and strengths of doppler/echo pulmonary artery systolic pressure-right heart catheterization correlations: a systematic literature review. *Echocardiography* .2015;32(1) : 10.1111/echo.12594 10-8 10.1111/echo.12594 24661140

[2] : Improving the echocardiographic assessment of pulmonary pressure using the tricuspid regurgitant signal-The "chin" vs the "beard". *Echocardiography* .2018;35(8) : 10.1111/echo.13893 1085-1096 10.1111/echo.13893 29676489

[3] : Correlation Between Doppler Echocardiography and Right Heart Catheterisation-Derived Systolic and Mean Pulmonary Artery Pressures: Determinants of Discrepancies Between the Two Methods. *Heart Lung Circ* .2021;30(5) : 10.1016/j.hlc.2020.10.009 656-664 10.1016/j.hlc.2020.10.009 33223493

# Response

**Published:** 2024-07-18

**Authors:** Ze Ming Goh

**Affiliations:** University of Sheffield, UK

The Authors demonstrated that MPA diameter and right atrial area measured on unenhanced CT scan have an acceptable to excellent diagnostic accuracy for predicting pulmonary hypertension detected on RHC.

 The Authors found that a MPA diameter ≥29 mm had the best sensitivity and specificity for noninvasively identifying patients with suspected pulmonary hypertension.

 The text is well written, each section is clearly presented, the statistics is adequate, the tables and figures are interesting and the discussion is complete.

 The strength of the manuscript is the proposal of a noninvasive technique, such as unenhanced CT scan, to predict pulmonary hypertension.

**I have some suggestions for the Authors:**

- In the Introduction section, there are some refusals: line 8, please delete the double “the” “the”.

**Response: ** We thank the reviewer for noticing the typographical error. We have now amended it: ‘Pulmonary hypertension is categorised into five major groups in the European Society of Cardiology (ESC)’

- In the test body please correct the refusals: 29mm and replace with 29 mm.

**Response:** We have made the correction.

- In the Methods section, please correct the refusal “imaiges” and replace with “images”.

**Response:** Thank you for noticing the typographical error. We have corrected it: ‘and the images were checked visually to ensure no contrast occurred.’

- In the Introduction section, the Authors could expand the limitations of transthoracic echocardiography in the systolic pulmonary artery pressure (sPAP) estimation.

Notably, sPAP may be underestimated: in the presence of pronounced enlargement of the effective area of the regurgitating tricuspid oriﬁce, which causes the reduction of tricuspid regurgitation velocity (TRV) and ‘truncation’ of the CW-Doppler spectrum of tricuspid regurgitation; in case of a small amount of tricuspid regurgitation leading to an incomplete Doppler spectrum, with consequent underestimation of SPAP, especially in patients with COPD and/or advanced lung disease.

 On the other hand, sPAP may not be accurate due to the frequent overestimation of right atrial pressure by echocardiographers.(Kyrianis)

 Other causes of TTE imprecision in estimating sPAP may be the following: female gender, arrhythmic cardiac electrical activity, systemic arterial hypertension, and diuretic treatment.

 With this regard, the Authors could cite and discuss the following references: Finkelhor *et al.* (2015 ^1^ ), Kyranis *et al.* (2018 ^2^ ) and Sonaglioni *et al.* (2021 ^3^ ).

**Response:** We thank the reviewer for the helpful comments and for providing the relevant references. We agree that it would be good to describe the limitations of transthoracic echocardiography in greater detail. We have added the following lines: 'Furthermore, accurate estimation pulmonary arterial pressure could be challenging on TTE. For example, systolic pulmonary arterial pressure (sPAP) may be underestimated in patients with pronounced enlargement of regurgitating tricuspid orifice, or patients with a small amount of tricuspid regurgitation, particularly in those with chronic obstructive lung disease (COPD) or other severe lung diseases. An overestimation of the right atrial pressure by echocardiographer could also lead to inaccurate estimation of sPAP. Other causes of inaccuracy include female gender, cardiac arrhythmia, systemic hypertension and diuretic.’ We have also removed the following line in the paragraph as we think it is not needed: 'Patients who experience dyspnoea, syncope and have the signs of right ventricular dysfunction should be investigated with a TTE'.

 In the Limitations section, the Authors could also mention that CT scan is limited by the ionizing exposition.

**Response:** We have added the following line to the limitations section: ‘The CT assessment was also limited by ionising exposition.’

# Reviewer report: Reviewer response for version 1

**DOI:** 10.21956/wellcomeopenres.18590.r46867

**Published:** 2021-11-15

**Authors:** Joseph Jacob

**Affiliations:** 1 Centre for Medical Image Computing, University College London, London, UK

The study evaluated measurements of structures on unenhanced CT to describe features seen in patients with pulmonary hypertension. The pilot study is well described and its clinical utility clear.

 Three measures (mPa, right atrial area and tricuspid annulus diameter) were identified as the best discriminators of pulmonary hypertension from Table 1. Yet the most significant result separating pulmonary hypertension from non-pulmonary hypertension patients was the left atrial area. This had comparable correlations to mean pulmonary artery pressure as the mPA diameter measurement. Interobserver variation was also satisfactory for this measurement. It would be interesting to see further evaluation of left atrial area in terms of ROC analyses.

 The remainder of the paper is well written with only minor typographical/grammatical errors.

Is the work clearly and accurately presented and does it cite the current literature?

Partly

If applicable, is the statistical analysis and its interpretation appropriate?

Partly

Are all the source data underlying the results available to ensure full reproducibility?

No source data required

Is the study design appropriate and is the work technically sound?

Yes

Are the conclusions drawn adequately supported by the results?

Yes

Are sufficient details of methods and analysis provided to allow replication by others?

Yes

Reviewer Expertise:

CT and CXR imaging of the lungs

I confirm that I have read this submission and believe that I have an appropriate level of expertise to confirm that it is of an acceptable scientific standard, however I have significant reservations, as outlined above.

# Response

**Published:** 2024-07-18

**Authors:** Ze Ming Goh

**Affiliations:** University of Sheffield, UK

The study evaluated measurements of structures on unenhanced CT to describe features seen in patients with pulmonary hypertension. The pilot study is well described and its clinical utility clear.

 Three measures (mPa, right atrial area and tricuspid annulus diameter) were identified as the best discriminators of pulmonary hypertension from Table 1. Yet the most significant result separating pulmonary hypertension from non-pulmonary hypertension patients was the left atrial area. This had comparable correlations to mean pulmonary artery pressure as the mPA diameter measurement. Interobserver variation was also satisfactory for this measurement. It would be interesting to see further evaluation of left atrial area in terms of ROC analyses.

**Response:** We thank the reviewer for the comments and agree that left atrial area demonstrated significant diagnostic accuracy in the current study. We have now included the following line in the discussion section: ‘Furthermore, left atrial area demonstrated comparable diagnostic accuracy (AUC 0.74 to 0.78) to right atrial area. This could possibly be explained by the inclusion of patients with pulmonary hypertension due to left heart disease in the study, as they are likely to have left atrial dilatation.’

 The remainder of the paper is well written with only minor typographical/grammatical errors.

**Response:** We have revised the manuscript for typographical and grammatical errors and corrected them.
